# Establishing Screening Programs for Presymptomatic Type 1 Diabetes: Practical Guidance for Diabetes Care Providers

**DOI:** 10.1210/clinem/dgaf194

**Published:** 2025-04-02

**Authors:** Steven B Leichter, Jamie L Felton, Cristy Geno Rasmussen, Patrick Rizzuto, Natalie Bellini, Osagie Ebekozien, Rifka Schulman-Rosenbaum

**Affiliations:** Piedmont Endocrinology Columbus, Columbus, GA 31904, USA; Department of Internal Medicine, Mercer University School of Medicine, Columbus, GA 31901, USA; Department of Pediatrics, Herman B Wells Center for Pediatric Research, Center for Diabetes and Metabolic Diseases, Indiana University School of Medicine, Indianapolis, IN 46202, USA; Implementation Science Department, Medstar Health Research Institute, Columbia, MD 21044, USA; Division of Endocrinology, Department of Pediatrics, Rutgers-Robert Wood Johnson Medical School, New Brunswick, NJ 08901, USA; Department of Medicine, Case Western Reserve University School of Medicine, Cleveland, OH 44106, USA; Diabetes and Metabolic Care Center, University Hospitals, Cleveland, OH 44124, USA; T1D Exchange, Boston, MA 02110, USA; Department of Population Health Science, John D Bower School of Population Health, The University of Mississippi Medical Center, Jackson, MS 39216, USA; Division of Endocrinology, Diabetes and Metabolism, Long Island Jewish Medical Center, Donald and Barbara Zucker School of Medicine at Hofstra/Northwell, Northwell Health, New Hyde Park, NY 11040, USA

**Keywords:** type 1 diabetes, autoimmune, autoantibody, screening, endocrinology

## Abstract

Type 1 diabetes (T1D) is an autoimmune disease with 2 presymptomatic stages (stages 1 and 2) that precede its clinical onset (stage 3). The presymptomatic stages of T1D are characterized by circulating autoantibodies that can be reliably detected by autoantibody screening panels. Identifying people in the presymptomatic stages of T1D can allow for monitoring and prevention of diabetic ketoacidosis. A disease-modifying therapy that has been shown to delay onset of stage 3 T1D is now also available for individuals with stage 2 disease, highlighting the importance of early detection. This intervention may delay the onset of stage 3 T1D. Updated guidance and protocols are needed to integrate autoantibody screening into standard practice. This report provides guidance for endocrinology providers on establishing clinical autoantibody screening programs within their practices, institutions, healthcare networks, and/or communities. Key steps include nominating a champion for the program, building a team to implement screening, and motivating other providers to participate. Implementation of screening requires standardizing several steps in the screening process, including communicating with individuals at risk, integrating screening into existing workflows, and streamlining logistics such as ordering and coding for autoantibody panels. Providers must have a plan to interpret and communicate results and to ensure that individuals may be appropriately followed in the future. Here, common barriers to screening are addressed, and practical solutions to facilitate the adoption and success of screening initiatives are offered.

Type 1 diabetes (T1D) is a progressive autoimmune disease that is often discovered upon presentation of clinical symptoms (1-3). However, before clinical onset (stage 3), there are presymptomatic stages (stages 1 and 2) that are characterized by the presence of circulating autoantibodies (AAbs) (4). Individuals with stage 1 T1D have positivity for 2 or more AAbs, are presymptomatic, and are normoglycemic. In stage 2 T1D, individuals have 2 or more AAbs and subclinical dysglycemia. By stage 3, symptoms and overt hyperglycemia are present, and individuals meet the standard diagnostic criteria for diabetes (4). Therefore, screening for T1D-associated AAbs is a reliable way to identify individuals with presymptomatic T1D prior to clinically significant beta cell loss and the need for exogenous insulin (4-6).

There are several potential benefits of AAb screening (Fig. 1). First, screening that leads to early detection of T1D gives clinicians an opportunity to intervene with disease-modifying therapy (DMT) (7). Teplizumab is the first DMT approved by the US Food and Drug Administration (FDA) that can alter the course of an autoimmune endocrine disease (8). Compared with placebo, teplizumab was shown to delay the onset (stage 3) of T1D by ∼2.7 years (9). The availability of teplizumab for people 8 years of age and older with stage 2 T1D is a significant development and has intensified discussions on the need for general population screening (10, 11).

**Figure 1.**
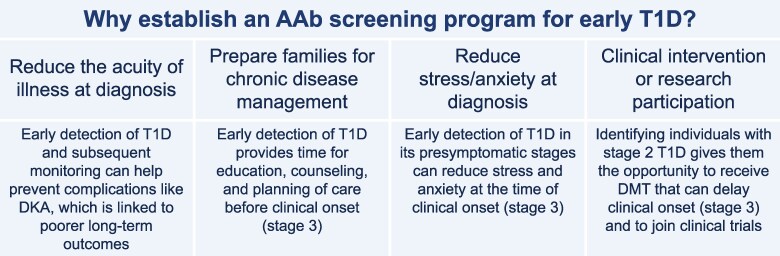
Benefits of establishing an AAb screening program for early T1D and how to identify individuals at risk for developing T1D.

Abbreviations: AAb, autoantibody; DKA, diabetic ketoacidosis; DMT, disease-modifying therapy; T1D, type 1 diabetes.

Early detection of T1D followed by metabolic monitoring can also result in an improved clinical presentation and a reduced risk of diabetic ketoacidosis (DKA) at or around the time of stage 3 diagnosis (11-15). DKA is a life-threatening condition that has short- and long-term impacts on health (16-18). The T1D Exchange and SEARCH for Diabetes in Youth registries reported that up to ∼40% of youth (<20 years of age) diagnosed with T1D initially presented with DKA in the United States (3, 19). An international retrospective study on DKA at diagnosis, including 10 European countries, reported DKA rates ranging from 21.6% to 43.8% (20). Risk of DKA depends on various factors. The rate of DKA can be higher in certain geographic regions, and it was exacerbated during the early waves of the COVID-19 pandemic (2, 21, 22). Data from the SEARCH for Diabetes in Youth study have shown that the prevalence of DKA at or near T1D diagnosis was highest in children aged 0 to 4 years (1, 3).

AAb screening reduces the rate of DKA around the time of diagnosis. The Autoimmunity Screening for Kids (ASK) study showed that although the DKA rate in Colorado was ∼60%, it could be as low as ∼5% in children who received AAb screening and metabolic monitoring (2, 11, 23). While most of the evidence around AAb screening and DKA rates at presentation is from pediatric studies, rates of DKA in adults are not insignificant (13). T1D Exchange registry data revealed that people 25 to 40 years of age showed a considerable rate of DKA at T1D diagnosis, ranging from 19% to 24% (19). An analysis of data from the ASK study found that AAb screening could be cost-effective if conducted in an area with high rates of DKA and with screening infrastructure, such as Colorado, and where avoidance of DKA leads to long-term improvements in glycosylated hemoglobin (HbA1c) (24).

Importantly, AAb screening can also provide people with presymptomatic T1D more time before symptom onset and insulin commencement to receive education and counseling. Diagnosis with T1D is life-altering and extremely anxiety-inducing for those affected and their families. Early detection that leads to better awareness of T1D prior to stage 3 diagnosis and the establishment of a care team and monitoring plan can reduce anxiety at the time of diagnosis (7, 25, 26).

Additionally, AAb screening is an important differential diagnostic tool. More than 40% of people over 30 years of age with new onset T1D are initially misdiagnosed with type 2 diabetes (T2D) (27-30). AAb testing can be used to appropriately diagnose individuals with T1D when they are not responding well to T2D therapies or if they do not fit the typical T2D phenotype (13, 27, 31, 32). Finally, AAb screening can give people the opportunity to take part in clinical trials (31).

Despite these advantages, AAb screening has mostly taken place within research-based screening programs and is done inconsistently in the clinical setting. This may be due to limited awareness among healthcare providers (HCPs) regarding the presymptomatic stages of T1D, appropriate tests to order including the existence of AAb panels, how to perform T1D screening, and the potential to reduce severity of disease at presentation. A lack of established workflow can also be a major barrier to AAb screening; therefore, screening processes that can be seamlessly integrated into busy practices must be developed (31).

This report aims to provide practical guidance for the T1D advocate and champion planning to establish a clinical AAb screening program within their practice, institution, health system, or community. We discuss considerations at fundamental steps, including building a team to plan the screening program, developing efficient processes, and motivating other providers to participate in the screening program.

A plain language summary of this work is available (33).

## Principles for Screening and Early Detection of T1D

In 1968, Wilson and Jungner proposed 10 principles for screening for the World Health Organization (WHO) (34). These principles can be used to identify the diseases for which screening programs could be used to benefit public health (34, 35). A recent status report by Sims and colleagues for the National Institute of Diabetes and Digestive and Kidney Diseases (NIDDK) T1D TrialNet Study group assessed these principles and their application to T1D screening (11). At the time of this publication, the second principle, which states, “*There should be an accepted treatment (…)*” had not been fully met, as FDA approval for teplizumab was still pending (10, 11). Although there is still no cure for T1D, the approval of teplizumab by the US FDA for delaying stage 3 onset in individuals with stage 2 T1D was an important step forward, and it strengthened the justification for screening programs (10, 11). Teplizumab is also marketed in the United Arab Emirates, Israel, and the Kingdom of Saudi Arabia. The European Medicines Agency (EMA) has accepted for review the regulatory submission for teplizumab to delay the onset of stage 3 T1D and also for early intervention in stage 3 (36).

## Who Should Be Considered for T1D Screening?

There is a lack of consensus on who should be screened for T1D. Although anyone can develop T1D, certain individuals are at increased risk (4). Individuals with a first-degree relative with T1D have a ∼15-fold higher risk of developing T1D than the general population (11, 37). Importantly, ∼90% of cases of new-onset T1D are in individuals without a first-degree relative with T1D (11, 38, 39). The prevalences of T1D in the general population and in those with first-degree relatives with T1D are ∼0.3% and ∼5%, respectively (37, 40, 41).

People with a personal or family history of other autoimmune conditions including celiac disease and autoimmune thyroid disease also have a higher risk of developing T1D compared with the general population (42-44). For example, in a study of several autoimmune conditions, risk of T1D was 2- to 3-fold higher in individuals with a parent with celiac disease, hypothyroidism, Addison's disease, pernicious anemia, primary biliary cirrhosis, rheumatoid arthritis, or systemic lupus erythematosus (45).

T1D can occur at any age. The detection of AAbs peaks between 9 months and 2 years of age, and the peak for clinical T1D diagnosis is at ∼10 years of age (among infants to 19-year-olds). While peak incidences occur at young ages, recent data indicate that more than half of new cases of T1D are in adults (46-48).

**Figure 2.**
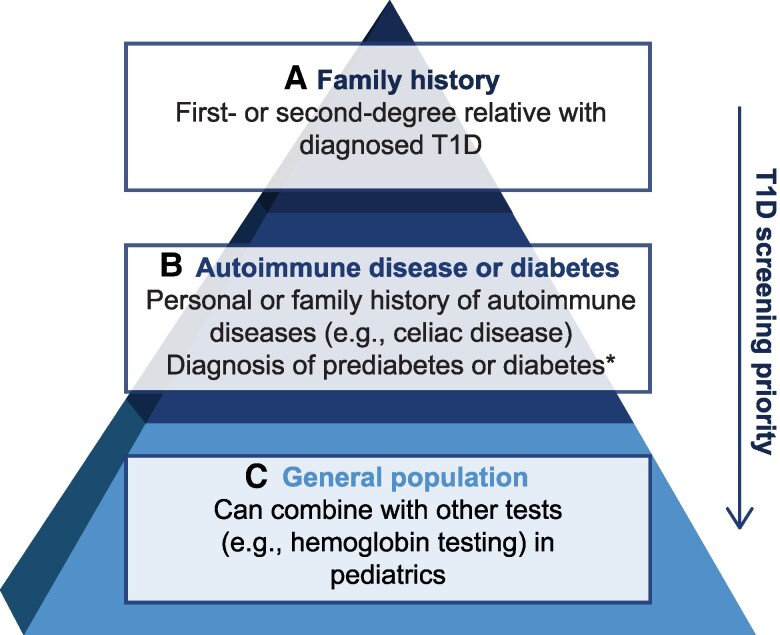
Suggested risk stratification approach for AAb screening programs for presymptomatic T1D. Using an approach to AAb screening that prioritizes individuals at increased risk for T1D may be pragmatic for clinics/institutions that do not have the infrastructure to screen all individuals. Individuals with a first-degree family member with T1D could be given the highest priority for screening, followed by those with second-degree relatives with T1D (A); followed by individuals with a personal or family history of other autoimmune diseases such as celiac, Hashimoto, Graves, or Addison disease, or those with a new prediabetes or diabetes diagnosis (B); followed by general screening of all individuals in the absence of an identified risk factor, and this could be done at certain ages or in parallel with other routine screening tests (C). Although this approach will fail to capture all individuals who will go on to develop T1D, establishing a tiered screening program may build infrastructure and pilot key processes that can be scaled up in the future. *For patients without typical features of T2D.

Abbreviations: AAb, autoantibody; T1D, type 1 diabetes; T2D, type 2 diabetes.

Approaches to screening that prioritize individuals at higher risk for T1D may be pragmatic for clinics that do not have the infrastructure to screen all individuals. In the approach shown in Fig. 2, individuals with a family history of T1D are given the highest priority for screening, with those having first-degree relatives given higher priority than those with second-degree relatives (49). This is followed by individuals with a personal or family history of other autoimmune diseases. In addition, clinical presentations of T1D and T2D can be misleading, and misdiagnoses are frequent. Therefore, patients who do not have typical features of T2D could also be screened for AAbs with medium priority (13). Although this approach will not capture most individuals who will go on to develop T1D, a tiered screening program may establish infrastructure and key processes that can be scaled up in the future. Importantly, family history-based screening fails to identify 90% of individuals that will develop clinical T1D (11, 50).

General population screening should be the ultimate goal to identify the majority of those that will develop T1D. This is being investigated and planned for implementation in select locations. The Fr1da study in Bavaria, Germany offered AAb screening to all children aged ∼2 to 5 years as a part of routine pediatrician visits, demonstrating feasibility of cross-sectional screening in pediatric care (26). In 2023, the Italian Parliament approved a law to introduce nationwide T1D and celiac disease screening for all children aged 1 to 17 years (51). If more DMTs for T1D become available, there may be a push toward more public health efforts such as those that include the general population. For example, the EarLy Surveillance for Autoimmune diabetes (ELSA) for children aged 3 to 13 years and Type 1 Diabetes Risk in Adults (T1DRA) studies are 2 general population T1D screening studies in the United Kingdom for children and adults, respectively (52). The EDENT1FI program will study the feasibility of general population screening for T1D and follow-up in several European countries with different healthcare settings (53).

There is much research and ongoing debate about the cost-effectiveness of general population screening. With improved screening methods and more treatments for prevention available, general population screening programs will expand, highlighting the importance of infrastructure development (50, 54).

## Roadmap for Establishing a T1D Screening Program

### Nominating a Program Champion

An important first step is to identify one or multiple “champion(s)” to lead the screening program. The champion is essential for acquiring the commitment of a busy clinical staff to implement the program, and can be a respected clinician, a diabetes educator, or an administrator who is involved with diabetes care. Irrespective of setting, the program champion should be highly knowledgeable about the screening process for early T1D. Key competencies are summarized in Table 1.

**Table 1.** Key competencies of a presymptomatic T1D AAb screening program champion and key resources to support these competencies

Key qualities of program champion	Key resources
**Likely a clinician, DE, or administrator within the practice in a leadership role who is:**	asktheexperts.org stopt1dprogram.org breakthrought1d.org/early-detection
*Knowledgeable about T1D and its presymptomatic stages, including likelihood of progression;*	
*Able to garner the support of other clinicians and a busy clinical staff to support the program, especially those who will be conducting screening;*	
*Able to establish efficient workflow processes for screening, communicating results, and further care to ensure adequate monitoring; AND*	
*Able to interpret and communicate test results*	

Abbreviations: AAb, autoantibody; DE, diabetes educator; T1D, type 1 diabetes.

### Consideration of Setting-Specific Challenges

HCPs conducting the screenings may be in private practices or large healthcare organizations, in primary care or specialty practices, and in urban or rural/regional environments. Health systems and healthcare delivery will vary depending on country and region. The unique workflow of the setting should be considered to identify obstacles that could impede the establishment of a screening program. Table 2 includes examples of different challenges that may arise along with some practical solutions.

**Table 2.** Potential challenges and practical solutions for establishing a screening program for presymptomatic T1D

Example challenge(s)	Practical solution(s)
1. Limited HCP awareness of presymptomatic stages of T1D and of AAb screening − resulting in limited screening performed by PCPs, endocrinologists, and other specialists outside of endocrinology	Foster education through lectures, grand rounds, “lunch and learn”-style sessions, materials shared through EHR, online resources (eg, “Ask the Experts”).
	**Education can encompass:**
	Presymptomatic stages of T1D
	Risk factors for T1D (family history, autoimmune disease)
	Potential benefits of screening (therapeutic intervention; more time for counseling and monitoring; DKA prevention; reduced long-term risk of complications)
	AAbs
	How to order AAb panels; research-based screening programs
	How to interpret AAb panel results
	How to communicate results with patients
	Referrals, next steps
2. AAb screening presents a workflow challenge − HCPs already have a busy patient load and cannot easily order panels	Ensure that there is a simple process for ordering an AAb panel within the host clinic/institution; eg, Quest Diagnostics and Labcorp both have panels that test all 4 AAbs. It should be as simple as a single action for any HCP who could participate in screening (eg, via EPIC or equivalent). Workflow solutions will be setting specific.
3. HCPs do not know how to interpret AAb panel results and what the next steps should be	Provide participating HCPs with resources, ensuring they are up-to-date with current consensus guidelines. Have a clearly developed process in place for further care, including referral to endocrinology when appropriate, for positive screens.
4. Family members of patients with T1D are not established patients and cannot be screened in the endocrinology office	If family members (eg, siblings) cannot be screened in the HCP's clinic/institution, provide patients with T1D and/or their caregivers a handout that explains familial risk for T1D, the benefits of AAb screening, links to resources, and available pathways to AAb screening. Involve PCPs and other specialists outside of endocrinology in screening individuals who are not under direct care in endocrinology.
5. No process in place to track and communicate results	Develop a streamlined process within clinic/institution for tracking test results and communicating results to the patient (eg, telehealth consult, in-person visit) that is within the capabilities of the clinical and administrative staff.

Abbreviations: AAb, autoantibody; DKA, diabetic ketoacidosis; EHR, electronic health record; HCP, healthcare provider; PCP, primary care provider; T1D, type 1 diabetes.

One major obstacle is the lack of awareness about the process for screening for presymptomatic T1D. Other obstacles might include difficulty in ordering the correct AAbs, a lack of administrative capability to track test results, and the absence of a clinician who is capable of interpreting and utilizing these results. Differences in practice settings will also impact resources and support for conducting screening (eg, method of blood draw, staff, electronic systems), follow-up procedures (eg, rescreening, monitoring), and communication/education tools.

The clinical setting also determines who can be screened (eg, adults vs children; those at high risk or the general population). For example, if a specialty diabetes education program is hosting the screening, only those enrolled in that program would be the targets of screening. If a hospital is hosting the screening, people who are seen by that hospital would be the targets. There may be some instances in which one organization that influences an entire community could be the host of the program. In those cases, the program needs to be designed to involve as many of the practitioners in that community as possible, and all of them would have to be educated in early T1D screening.

### Building a Team to Plan the Screening Program

A team will need to be established to plan and ultimately advocate for the screening program. Providers from disciplines and settings who would be involved in conducting the screenings would be valuable members of the team.

For example, endocrinologists and primary care providers (PCPs) (ie, pediatricians, family practitioners, and internal medicine physicians) can offer care to family members of their patients with T1D, but PCPs will be most crucial for screening of the general population. Individuals at risk for developing T1D are likely not being followed by an endocrinologist. Therefore, it is important that PCPs are included in any screening team, and that efforts are made toward raising their awareness of early T1D and of screening.

In the United States and Europe, PCPs currently screen for other metabolic conditions, so it is feasible that they can also play a crucial role in T1D screening (13). Non-diabetes specialists, such as gastroenterologists, see people with non-endocrine autoimmune diseases (eg, celiac disease) who may have a higher risk of developing T1D (55). To capture people outside of an endocrinology practice for screening, it will be important to include providers from other specialties on the team. Both PCPs and specialists could contribute valuable insights on how to implement screening processes within their specific clinical environments and how to motivate their respective colleagues to take part in the screening program.

### Developing Processes for the Screening Program

When establishing T1D screening processes, it is important to consider how they can be streamlined and built into existing workflows, as specialists and PCPs may report feeling like they are already working at their full capacity with a busy patient load. Several components of the program can be standardized, including communication with patients, ordering electronic health record (EHR)-preferred AAb panels, timing of screening, diagnostic codes, follow-up procedures, monitoring, and communication with endocrinology with positive results for support and/or referral.

#### Communicating with at-risk individuals about AAb testing

For several reasons, some people may not want to know their AAb status. For example, they may have anxiety about the potential for a positive result or feel that they are capable of recognizing the signs and symptoms of T1D before becoming critically ill (56, 57). It is important to educate people at increased risk for T1D and/or their caregivers about the benefits of screening and how it can be a proactive measure to improve long-term health (56, 57).

While validating the patient's concerns about the potential for a positive AAb test result, the provider should emphasize studies that demonstrate that early detection often results in less distress and better health around the time of T1D diagnosis, compared with finding out about T1D when symptoms manifest (25, 26, 57). It is necessary to explain that T1D is an autoimmune disease and to outline the differences between T1D and T2D, as these are commonly confused. Misunderstandings among people with T1D and families surrounding T1D vs T2D can lead to stigma, blame, and misconceptions that T1D can be treated with lifestyle changes alone (56). After providing the patient with clear information about T1D and AAb screening, it is important to obtain their informed consent prior to ordering the AAb panel(s). If, after discussion, patients or their caregivers remain skeptical of screening, providing written information for review and deferring further discussion and a decision to a later time is appropriate.

Where applicable, HCPs can communicate with people about the costs of screening (31). Costs may be covered by private or public health insurance programs, but if not, they could be considerable. In addition, for patients who have participated in research screening programs, it may be necessary to clarify the costs if they transition to receiving care in a non-research clinical setting. Screening can be done at no cost as part of research studies such as TrialNet, which is available to first-degree relatives aged 2 to 45 years in the United States, Canada, Europe, and Australia. The ASK Research Program also allows people to be part of research and offers screenings at no cost to children and adults in the United States with or without a family history of T1D. AAb screening programs in Europe include the ELSA and T1DRA studies in the United Kingdom, and EDENT1FI (52, 53).

Diabetes specialists and PCPs may consider discussing AAb screening of family members with their patients with T1D. Those interested can be provided with informational handouts to share with their family members. The handouts can describe the options for screening and can inform about the benefits of early detection. To reduce time in documenting the discussion, HCPs can create dot phrases (aka macros, quick phrases, or smart phrases) in EHR.

#### Ordering AAb tests

Screening for presymptomatic T1D involves testing for specific AAbs against glutamic acid decarboxylase (GAD), insulin (IAA), islet antigen-2 (IA-2), and zinc transporter 8 (ZnT8) (4). Ordering AAb tests will need to be simplified, by eliminating any obstacles that prevent relevant providers from being able to easily order an AAb panel. In many centers, this panel is only an option for specialized providers such as endocrinologists. Since many screening programs will involve PCPs and other providers, they also need to be able to order AAb panels without adding additional time and burden to their usual workflow.

Providers conducting AAb screening through an external lab should be aware that standard panels may not include all the specific AAbs by default. As an example, the Quest Diagnostics autoimmunity panel for T1D until recently only included GAD, IAA, and IA-2, but not ZnT8, which needed to be requested separately. In a triumph for AAb screening, the Quest Diagnostics system was recently updated (test code: 13621), which has streamlined the process of ordering the 4-AAb panel for busy clinicians. The Labcorp autoimmunity panel for T1D (test code: 504050) also includes all 4 specific AAbs (Table 3).

**Table 3.** **Order codes or source of T1D AAb panels and screening kits**.***^a^***

Panel description	Order # / Source
*Quest Diagnostics*	
GAD, IAA, IA-2, ZnT8	13621
ICA screen with reflex to titer	36741
*Labcorp*	
GAD, IAA, IA-2, ZnT8	504050
Antipancreatic islet cells	160721
*Enable Biosciences*	
GAD, IAA, IA-2	https://type1testing.enablebiosciences.com/

Abbreviations: GAD, glutamic acid decarboxylase; IA-2, islet antigen-2; IAA, insulin autoantibody; ICA, islet cell antibody; T1D, type 1 diabetes; ZnT8, zinc transporter 8.

^a^Order numbers may vary and are provided as an example for reference only. It is the responsibility of the provider ordering the tests to verify the correct order numbers.

If testing for all 4 AAbs initially is not feasible or practical in a given country or region, screening should be streamlined to include 3 AAbs in a single panel. For example, testing for ZnT8 may not be covered by public health insurance in certain regions. This approach may result in not capturing a proportion of individuals with single AAb positivity. A study of people with recent-onset T1D showed that 26% of those considered AAb negative when testing for GAD, IAA, and IA-2 were positive for ZnT8 (58). In addition, a recent systematic review found that the presence of certain AAbs may influence risk of progression to T1D (59). In people with single AAb positivity, the presence of ZnT8 was associated with a higher risk of progression to T1D than in those with single AAb positivity for GAD, IAA, or IA-2 (59, 60). In addition, ZnT8 may be a useful marker in older individuals, as IAA levels can decline with age (58).

A screen for islet cell autoantibodies (ICAs) is one of the original assays for T1D-associated autoimmunity that has been and continues to be used in the research setting but is less available in clinical practice. ICAs are measured by an indirect immunofluorescence assay on pancreatic tissue and can detect multiple undefined antigens (13). Although ICAs can assist in risk determination, confirmation testing with “biochemical” AAbs (GAD, IAA, IA-2, and ZnT8) is recommended in the American Diabetes Association (ADA) Standards of Care and Breakthrough T1D (formerly JDRF) consensus guidance document (4, 13).

#### When to screen

AAb screening can be done at any age, ideally beginning as early as 9 months. At this time, maternal antibodies may be circulating and may include AAbs if the mother has undiagnosed early T1D or misdiagnosed gestational diabetes. Importantly, T1D can occur before the age of 1 year, and younger age at seroconversion is a predictor of more rapid T1D progression and higher risk for DKA at diagnosis (5, 46, 61). It is efficient to combine AAb testing with other routine screening tests. For example, in the United States, T1D screening in pediatrics may be combined with lead or hemoglobin testing (1-2 years of age), preschool vaccinations (4-6 years of age), and lipid screening (9-11 years of age) (61-63). EHRs often feature customizable order sets, which, after modification, may allow providers to add AAb testing to other routine screening with no additional steps.

One analysis reported that screening at 2 years and 6 years of age can capture children who will progress to have clinical T1D by 15 years of age with 82% sensitivity (64). The most cost-effective and pragmatic approach to screening all children may be for pediatricians to target these age ranges for screening, while diabetes specialists can capture people who have not already been screened as part of routine visits in primary care. As screening programs in certain regions evolve to include the general population, genetic screening for T1D risk in newborns can be combined with AAb screening at older ages (11, 65). Existing screening programs for early T1D are described in Table 4.

**Table 4.** Examples of screening programs for T1D

Screening program	Description
**Autoimmunity Screening for Kids (ASK)** www.askhealth.org	Research screening program offering screening at no cost to all children (ages 1-17) and adults in the US to detect T1D and celiac disease.
**Combined Antibody Screening for Celiac and Diabetes Evaluation (CASCADE)** www.cascadekids.org	T1D and celiac disease research study screening for children born in the state of Washington. Study enrolling children ages newborn to 8 months or 4 to 8 years. No cost.
**Early Check T1D** https://earlycheck.org/	Early Check is a research study that uses newborn blood samples to test for genetic risk for several conditions, including T1D.
**EarLy Surveillance for Autoimmune Diabetes (ELSA)** https://www.elsadiabetes.nhs.uk/about	Research screening program that is exploring the feasibility and benefits of screening for T1D. ELSA offers T1D screening for children aged 3-13 years.
**European action for the Diagnosis of Early Non-clinical Type 1 diabetes For disease Interception (EDENT1FI)** https://www.edent1fi.eu/	Collaborative study that aims to comprehensively screen for and detect T1D in its presymptomatic stages, develop strategies for preventative and disease-modifying therapies, explore the use of validated biomarkers for early T1D progression, inform the public, HCPs, and authorities about new T1D paradigms, and effectively manage presymptomatic T1D to reduce disease impact at clinical onset.
**Population Level Estimate of type 1 Diabetes risk Genes in Children (PLEDGE)** https://research.sanfordhealth.org/fields-of-research/diabetes/pledge	Research screening for T1D and celiac disease; Sanford Health patients under 6 years of age and from 9-16 years of age are eligible. Children aged 6-17 years with a sibling with T1D or T1D AAbs are also eligible. The PLEDGE Study has clinics across North Dakota, South Dakota, Minnesota, Iowa, and Nebraska.
**TrialNet** www.trialnet.org	International network for T1D research offering risk screening for relatives of people with T1D or people who have tested positive for at least one T1D-associated antibody outside of TrialNet. Options for clinic, lab, and at-home testing.
**Type 1 Diabetes Risk in Adults (T1DRA)** https://t1dra.bristol.ac.uk/	Research screening program offering T1D screening to adults in the UK aged 18-70.

Abbreviations: AAb, autoantibody; T1D, type 1 diabetes; UK, United Kingdom; US, United States.

#### Diagnostic codes

The International Classification of Diseases, 10th revision (ICD-10) codes **Z13.1** (encounter for screening for diabetes mellitus) or **Z83.3** (family history of diabetes mellitus) can be used for screening individuals with a family history of T1D. The code **Z13.29** (encounter for screening for other suspected endocrine disorder) can be used for screening individuals with a personal or family history of autoimmune diseases. After being proposed by T1D advocates and experts, new ICD-10 codes for presymptomatic T1D became available on October 1, 2024. These include **E10.A0** (type 1 diabetes mellitus, presymptomatic, unspecified), **E10.A1** (type 1 diabetes mellitus, presymptomatic, stage 1), and **E10.A2** (type 1 diabetes mellitus, presymptomatic, stage 2) (66). These codes will be helpful for monitoring visits after a patient has screened positive for AAbs. Diagnostic and Current Procedural Terminology (CPT^®^) codes are listed in Table 5 and Table 6, respectively.

**Table 5.** ICD-10 diagnostic codes for presymptomatic T1D

Description	Code*^a^*
**Screening**	
Encounter for screening for diabetes mellitus	Z13.1
Family history of diabetes mellitus	Z83.3
Family history of other endocrine, nutritional, and metabolic diseases	Z83.49
Encounter for screening for other suspected endocrine disorder	Z13.29
Endocrine disorder, unspecified	E34.9
Hyperglycemia, unspecified	R73.9
**Monitoring**	
Type 1 diabetes mellitus, presymptomatic, unspecified	E10.A0
Type 1 diabetes mellitus, presymptomatic, stage 1	E10.A1
Type 1 diabetes mellitus, presymptomatic, stage 2	E10.A2
**T1D diagnosis**	
Type 1 diabetes mellitus	E10.1-E10.9*^b^*

Abbreviations: ICD-10, International Classification of Diseases, 10th revision; T1D, type 1 diabetes.

^a^Codes may vary and are provided as an example for reference only. It is the responsibility of the provider to verify the correct codes.

^b^Further specifications based on complications.

**Table 6.** **CPT^®^ codes for AAb panels and blood glucose testing**.***^a^***

Description	Code
**AAbs**	
GAD, IA-2, ZnT8	86341
ICA	
IAA	86337
**Blood glucose**	
Glucose tolerance test (GTT), 3 specimens (includes glucose)	82951
Glucose, quantitative, blood (except reagent strip)	82947
Glucose post-glucose dose (includes glucose)	82950
Hemoglobin glycosylated (HbA1c)	83036

Abbreviations: AAb, autoantibody; CPT, Current Procedural Terminology; GAD, glutamic acid decarboxylase; IA-2, islet antigen-2; IAA, insulin autoantibody; ICA, islet cell autoantibodies; ZnT8, zinc transporter 8.

^a^Codes may vary and are provided as an example for reference only. It is the responsibility of the provider to verify the correct codes.

#### Processes following positive/negative screening test results

A plan for appropriate care and follow-up after screening results are received is critical to any successful screening program. Since the approval of teplizumab, consensus guidelines for monitoring individuals after antibody screening have been published by the American Diabetes Association, the International Society for Pediatric and Adolescent Diabetes (ISPAD), and Breakthrough T1D, and are listed in Table 7. These resources describe follow-up screenings (for negative tests), confirmation of screening results (for positive tests), metabolic monitoring of people with positive tests, and recommended communication with patients/caregivers. All relevant staff, including clinical and support staff, should be trained in the screening process and should know what discussions need to take place following screening. For individuals with positive autoimmune profiles, the institution where the screening program is taking place will determine the desired patient flow either within the organization or community.

**Table 7.** Guidelines and resources on screening and monitoring for T1D

Resource	Description
**American Diabetes Association (ADA) Standards of Care in Diabetes—2025** 1. Improving Care and Promoting Health in Populations https://diabetesjournals.org/care/article/48/Supplement_1/S14/157553/1-Improving-Care-and-Promoting-Health-in 2. Diagnosis and Classification of Diabetes https://diabetesjournals.org/care/article/48/Supplement_1/S27/157566/2-Diagnosis-and-Classification-of-Diabetes 3. Prevention or Delay of Diabetes and Associated Comorbidities https://diabetesjournals.org/care/article/48/Supplement_1/S50/157550/3-Prevention-or-Delay-of-Diabetes-and-Associated	Clinical practice recommendations developed by the American Diabetes Association Professional Practice Committee. Updated in 2025 (4, 67, 68).
**Barbara Davis Center for Diabetes “Ask the Experts”** www.asktheexperts.org	Program for HCPs and people who have screened positive for T1D-associated AAbs and their families in the United States. Provides education and support, monitoring resources and guidance, and access to early treatment interventions. Information available for providers and families.
**Breakthrough T1D (formerly JDRF)** https://pubmed.ncbi.nlm.nih.gov/38912694/ Phillip et al, (2024) (13)	Consensus guidelines on monitoring individuals who have tested positive for at least one T1D-associated AAb. Addresses partnerships between specialists and primary care, confirming positive tests, monitoring, trial participation and therapy, education, and unmet need in the early stages of T1D. Published simultaneously in *Diabetes Care* and *Diabetologia* in 2024 (13).
**Historical Insights and Current Perspectives on the Diagnosis and Management of Presymptomatic Type 1 Diabetes** https://pubmed.ncbi.nlm.nih.gov/37695674/ Simmons et al, (2023) (61)	Guidance for interpreting and confirming T1D AAb screening results and follow-up of individuals with presymptomatic T1D. A modified Delphi method was used to identify consensus (61).
**International Society for Pediatric and Adolescent Diabetes (ISPAD) Clinical Practice Consensus Guidelines 2024** https://pubmed.ncbi.nlm.nih.gov/39662065/ Haller et al, (2024) (50)	Clinical recommendations for children, adolescents, and young adults with diabetes worldwide. Provides an updated summary of recommendations for screening for T1D and monitoring for people with early-stage T1D (50).
**Recommendations for Screening and Monitoring the Stages of Type 1 Diabetes in the Immune Therapy Era** https://pubmed.ncbi.nlm.nih.gov/39011423/ Moore et al, (2024) (7)	Recommendations for PCPs and pediatricians to understand the rationale behind identifying individuals with presymptomatic T1D and guidance for screening and monitoring (7).
**Screen TO Prevent (STOP) T1D** https://www.stopt1dprogram.org/	Support for HCPs, individuals, and families to find information about screening programs, monitoring, and available clinical trials. Patients can join the STOP T1D registry.

Abbreviations: AAb, autoantibody; HCP, healthcare provider; JDRF, Juvenile Diabetes Research Foundation; PCP, primary care provider; T1D, type 1 diabetes.

Accurate and timely discussion of the clinical implications of screening results with patients and family is essential. It may be important to explain to patients the risk associated with single vs multiple autoantibody positivity. One study estimated that the risk of children progressing to T1D after 10 years following a positive test for a single AAb is ∼14.5% and following a positive test for multiple AAbs is ∼69.7%, with the risk being even higher in children who test positive under the age of 3 years (5). Discussing this before screening occurs may help prevent undue distress in people who screen positive for a single antibody if results are viewable before discussion with their physician. T1D also can progress more rapidly in younger children with multiple AAbs (46). When there is confirmed positivity for T1D AAbs, providers may refer patients to a specialist in T1D for further education, metabolic monitoring, consideration of DMT, and opportunities to participate in clinical research (67).

It will be important to consider cultural factors that could influence how patients and their families perceive an AAb screen result or a T1D diagnosis. A study on the perceptions and experiences of Hispanic adolescents in the United States with T1D reported some culturally specific themes, including a higher emphasis on familial well-being, parental concerns about cultural foods exacerbating T1D, and the positive influence of having a Spanish-speaking member of their healthcare team (69).

### Educating and Motivating Providers to Participate in the Screening Program

The final step in implementing the screening program is to raise awareness of the screening program and to engage providers to participate. Education is an important part of motivating HCPs to take part. In its simplest form, education can occur through conversations with colleagues to raise awareness of early-stage T1D and the screening program.

Since many providers may be unfamiliar with early-stage T1D, advocates for screening will need to use multiple layers of education (eg, grand rounds, department-specific meetings, lectures, or “lunch and learn” sessions) to educate colleagues. Information about early-stage T1D and the screening program could then be shared through organizational emails or newsletters. Resources that concisely describe the stages of T1D and processes for screening and monitoring for T1D tailored to local or regional system (eg, order sets, specialists) could also be embedded within the integrated EHR system. EHR systems could also provide alerts notifying providers of opportunities to screen specific patients, including children at certain ages along with other routine screenings, people with a family history of T1D, or people with a personal or family history of other autoimmune diseases. To ensure proper identification of those at highest risk, there needs to be a concerted effort to document family history of T1D in EHRs (57).

**Figure 3.**
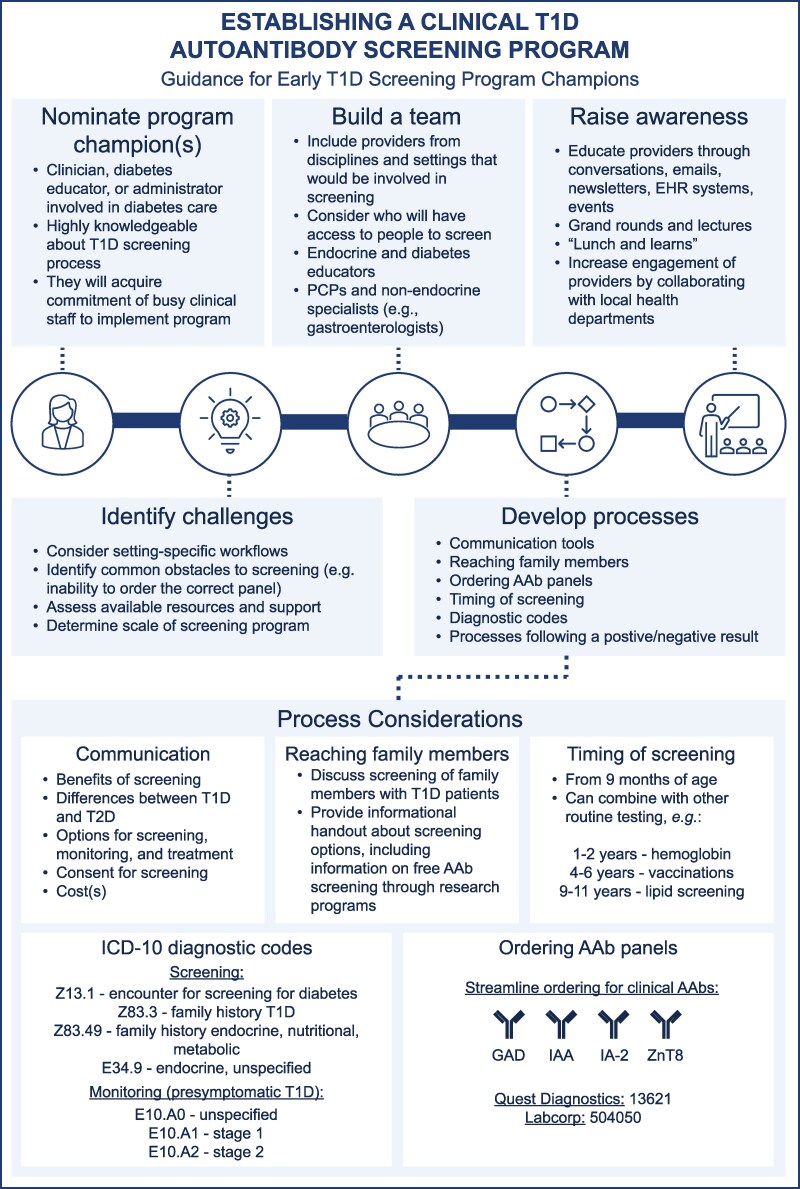
Establishing a clinical AAb screening program for early T1D: guidance for endocrinology providers.

Abbreviations: AAb, autoantibody; EHR, electronic health record; GAD, glutamic acid decarboxylase; IA-2, islet antigen-2; IAA, insulin autoantibody; ICD-10, International Classification of Diseases, 10th revision; PCP, primary care provider; T1D, type 1 diabetes; T2D, type 2 diabetes; ZnT8, zinc transporter 8.

For community-wide screening programs, educational events such as presentations at conferences can be held for appropriate providers to promote awareness of early-stage T1D and the screening program. It may also be beneficial to have the support of local public health departments to increase engagement of other providers. Ultimately, screening programs will have the broadest impact if they are designed to be integrated into many healthcare organizations and practices in a community. Key steps for establishing a clinical AAb screening program are summarized in Fig. 3.

## Communicating the Value of Screening to Decision-Makers

It will be important to communicate the value of the screening program when engaging with administrators and decision-makers, as support from the participating institution or from healthcare organizations will be instrumental to the success of any program. Tracking metrics will be useful in maintaining continued support within an institution or healthcare organization. These metrics could include how a screening program and early detection of T1D can reduce DKA and improve clinical outcomes (2, 12, 23). Metrics tied to cost may be valuable for future cost-benefit analyses. The opportunity to offer a novel DMT can also be emphasized to gather institutional support.

To support wide-scale efforts to screen for early T1D, advocates can communicate with local, state, or federal legislators to provide funding for early T1D screening. An effective presentation or letter could outline the avoidable burden of acute and potentially life-threatening complications such as DKA, provide a concise summary of the benefits of screening, tell a story about a specific family that benefited, and conclude with a request for a specific action from the legislator. It would be helpful to use visual aids and to leave a one-page summary of the key points with the legislator as a reference. It is customary to follow up after the meeting to thank the legislator and/or staff for their time and to reiterate the key messages and requests (70).

## Conclusions

Screening for presymptomatic T1D is important for identifying individuals when they have stage 1 or stage 2 T1D to reduce the risk of DKA prior to T1D diagnosis. With the opportunity for intervention with DMT, there is even greater incentive to build screening processes into standard of care. How diabetes advocates and champions design a clinical screening program depends on variables such as the clinical setting, the available resources, and the populations to be screened. Including the input of PCPs and specialists will be instrumental to a successful screening program. Essential steps when building the infrastructure for a screening program include developing processes that can be efficiently incorporated into existing workflows and motivating other providers to participate through outreach and education. Finally, health systems will need to work toward general population screening to effectively diagnose T1D in the early stages in most cases (11).

## Data Availability

Data sharing is not applicable to this article as no datasets were generated or analyzed during the current study.

## Abbreviations

AAbs: autoantibodies
ASK: Autoimmunity Screening for Kids
DKA: diabetic ketoacidosis
DMT: disease-modifying therapy
GAD: glutamic acid decarboxylase
EHR: electronic health record
FDA: Food and Drug Administration
HbA1c: glycosylated hemoglobin
HCP: healthcare provider
IA-2: islet-antigen 2
IAA: insulin autoantibody
ICA: islet cell autoantibody
PCP: primary care provider
T1D: type 1 diabetes
T2D: type 2 diabetes
ZnT8: zinc transporter 8
